# A low-cost method for visible fluorescence imaging

**DOI:** 10.1107/S2053230X17015941

**Published:** 2017-11-10

**Authors:** Crissy L. Tarver, Marc Pusey

**Affiliations:** a Department of Biological Science, University of Alabama in Huntsville, Huntsville, AL 35899, USA

**Keywords:** fluorescence, trace fluorescent labeling, low-cost imaging, protein crystallization

## Abstract

A wide variety of crystallization solutions are screened to establish conditions that promote the growth of a diffraction-quality crystal. Screening these conditions requires the assessment of many crystallization plates for the presence of crystals. Automated systems for screening and imaging are very expensive. A simple approach to imaging trace fluorescently labeled protein crystals in crystallization plates has been devised, and can be implemented at a cost as low as $50. The proteins β-lactoglobulin B, trypsin and purified concanavalin A (ConA) were trace fluorescently labeled using three different fluorescent probes: Cascade Yellow (CY), Carboxyrhodamine 6G (CR) and Pacific Blue (PB). A crystallization screening plate was set up using β-lactoglobulin B labeled with CR, trypsin labeled with CY, ConA labeled with each probe, and a mixture consisting of 50% PB-labeled ConA and 50% CR-labeled ConA. The wells of these plates were imaged using a commercially available macro-imaging lens attachment for smart devices that have a camera. Several types of macro lens attachments were tested with smartphones and tablets. Images with the highest quality were obtained with an iPhone 6S and an AUKEY Ora 10× macro lens. Depending upon the fluorescent probe employed and its Stokes shift, a light-emitting diode or a laser diode was used for excitation. An emission filter was used for the imaging of protein crystals labeled with CR and crystals with two-color fluorescence. This approach can also be used with microscopy systems commonly used to observe crystallization plates.

## Abstract 2

A very low cost method for carrying out imaging of trace fluorescently labeled protein crystallization plates is described.

## 1. Introduction   

The structure and function of many proteins are often determined by X-ray crystallography, which requires crystals with high regularity. Crystallization conditions that facilitate the growth of a single crystal that diffracts to atomic resolution must be ascertained by screening an assortment of solutions. Screening conditions involves setting up multiple plates of trial conditions and then periodically reviewing these conditions for the growth of crystals. The reviewing process may be carried out manually using a microscope, or it may be performed using an automated plate-imaging system. There are two major difficulties in the plate-reviewing process: distinguishing macromolecule crystals from salt crystals and the actual detection of macromolecule crystals. Automated imaging systems address these difficulties by focusing the detection approach on characteristics that are likely to be present in macromolecules but not small molecules, such as the presence of tryptophan. Imaging approaches based on recognizing crystals, typically an extensive process, have some of the same difficulties as direct visual observation. Most recently developed methods now use fluorescence or some other optical manipulation to clearly reveal the presence of macromolecule crystals.

An early approach was to make use of the intrinsic fluorescence in macromolecules (Judge *et al.*, 2005 ▸; Dierks *et al.*, 2010 ▸) arising primarily from the presence of the amino acid tryptophan. The use of UV is not recommended for direct microscopy visualization. Two-photon fluorescence has been used with an excitation wavelength of 515 nm (Madden *et al.*, 2011 ▸). This method requires scanning a high-intensity light source through a region of interest, using a high numerical aperture lens to achieve a sufficiently high photon density that fluorescing molecules can absorb two photons, at twice the wavelength of the normal absorption, within 10^−15^–10^−16^ s of each other (Xu & Webb, 1997 ▸). This requires a high-power light source plus a means of scanning the light through the sample and acquiring the emission signal from each scanned point. Another approach is the use of second-order nonlinear imaging of chiral crystals, also known as SONICC (Kissick *et al.*, 2010 ▸). A different fluorescence approach is based upon visible-light excitation of native fluorescence (Lukk *et al.*, 2016 ▸). The fluorescence is apparently not chromophore-dependent, but is believed to be based on conjugated double bonds and electron delocalization from crystal packing. The fluorescence can be obtained over a range of visible wavelengths, but was found to be strongest with excitation at <405 nm, with a rather wide Stokes shift (excitation at ∼405 nm, emission at ∼485 nm). The fluorescence dramatically increased with decreasing temperature and varied from protein to protein. Some proteins were found to not fluoresce. A relatively low-cost approach (<1000 Euro) has been described by Watts *et al.* (2010 ▸). This approach is based on the increase in fluorescence when a soluble probe, in this case 1-anilinonaphthalene-8-sulfonic acid (1,8-ANS), binds to hydrophobic regions, as described by Groves *et al.* (2007 ▸).

The trace fluorescent labeling (TFL) approach was, as with the above methods, introduced to speed up and simplify the process of finding macromolecule crystals in screening plates and to differentiate macromolecule crystals from salt crystals (Forsythe *et al.*, 2006 ▸). Relatively simple instrumentation can be used, and the technique is not dependent upon the presence of fluorescing amino acids as the fluorescent probe is covalently bound to the protein. The safety issues associated with UV fluorescence are avoided, and one can choose from a range of colors when using visible fluorescence. The labeling method is simple and takes about 15 min to carry out, and the target labeling level of 0.1–0.5% is achieved if it is performed according to protocol (Pusey *et al.*, 2015 ▸). Labeling at this level has been shown to not affect the nucleation rate or the diffraction quality of the crystals obtained (Forsythe *et al.*, 2006 ▸).

The primary barrier to the use of all fluorescence-based methods is the cost of the required instrumentation. Relatively low-cost fluorescence microscopy systems can be obtained; however, they are more suited for looking at slide-mounted specimens instead of crystals in thicker and larger crystallization plates. While fluorescence may be the most unambiguous means of identifying macromolecule crystals and crystallization conditions, it is not readily available to smaller structural biology research groups or students. Here, we present a simple and low-cost approach to visible fluorescence imaging of TFL crystallization screening plates.

## 2. Materials and methods   

### 2.1. Proteins   

The proteins used throughout this work were trypsin (Sigma, catalog No. T1426), β-lactoglobulin B (Sigma, catalog No. L8006) and concanavalin A (ConA). ConA was purified in-house from jack beans using the method of Agrawal & Goldstein (1967 ▸). The purified ConA was stored in crystalline form at 4°C. An aliquot of the precipitate was removed, concentrated by centrifugation, dissolved in weak ammonium hydroxide solution and underwent buffer exchange using a centrifugal desalting column into the crystallization buffer 0.025 *M* Na HEPES, 0.025 *M* NaCl pH 7.5.

### 2.2. Crystallization   

Each protein was trace fluorescently labeled (TFL) as described previously (Pusey *et al.*, 2015 ▸) using either Cascade Yellow (CY; Invitrogen, catalog No. C-10164), Carboxy­rhodamine 6G (CR; Invitrogen, catalog No. C-6157) or Pacific Blue (PB; Invitrogen, catalog No. P-10163). Crystallization plates were set up using (i) CY-labeled ConA, (ii) CR-labeled ConA, (iii) PB-labeled ConA, (iv) a mixture containing 50% PB-labeled ConA and 50% CR-labeled ConA, (v) CR-labeled β-lactoglobulin B and (vi) CY-labeled trypsin. The sitting-drop vapor-diffusion method was used for all crystallizations. ConA was crystallized using the JCSG-*plus* screen (Molecular Dimensions, catalog No. MD1-37) in Corning CrystalEX plates (Hampton Research, catalog No. HR8-140). Each of the 96 reservoirs was filled with 50 ml precipitant solution, and the three wells were prepared at protein:precipitant ratios of 1:1, 2:1 and 1:2. All plates were stored at room temperature. Trypsin was crystallized using the conditions given on the Rigaku website (http://www.rigaku.com) and β-lactoglobulin B was crystallized from 0.1 *M* sodium citrate, 3.0 *M* ammonium sulfate pH 4.0.

### 2.3. Fluorescent imaging   

**Figure 1:**
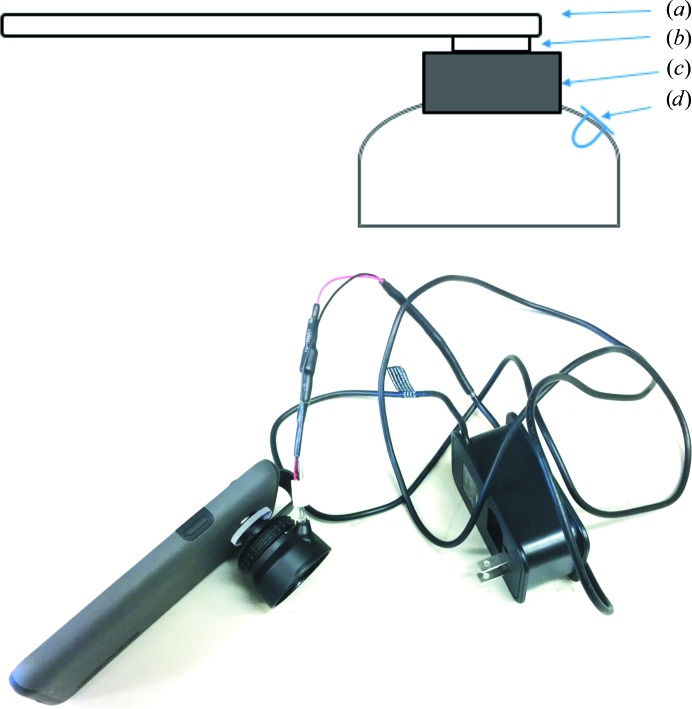
Top: diagram of the cell-phone-based fluorescence imaging system. (*a*) Cell phone; (*b*) emission filter; (*c*) 10× macro lens with attached hood; (*d*) LED mounted in lens hood. Not shown are the aluminium tube used to hold and aim the LED, the LED power supply and the clip that holds the assembly to the cell phone. Bottom: image of the complete cell-phone-based system, showing the macro lens with black hood attached to a Samsung Galaxy cell phone. Also shown are the power supply and the connections to the LED.

**Figure 2:**
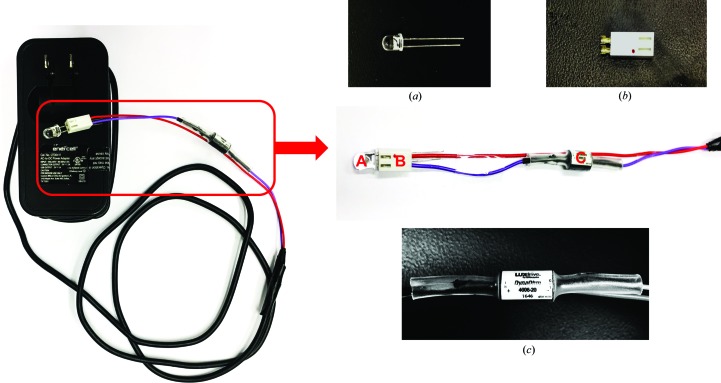
Complete LED power supply and the components used in assembling this light source. The LED bulb, the wiring connector that the LED plugs into and the LED current-limiting resistor are labelled A, B and C, respectively, and the individual components are shown in (*a*), (*b*) and (*c*).

The imaging lens used was an AUKEY Ora 10× macro lens purchased from Amazon (Fig. 1 ▸). The lens clips onto the camera of a smartphone or tablet, and it comes with a hood. The hood was modified by epoxying a short piece of 5 mm internal diameter aluminium tubing to a hole drilled in the top of the hood, near to where it attaches to the lens assembly. When applying the epoxy, the aluminium tubing was aimed to the center of the hood by a second piece of aluminium tubing with a 5 mm outer diameter to indicate the path of the excitation beam. After the epoxy had set, the exterior of the hood was covered with a coating of flat black paint. The excitation source used to screen crystallization plates containing protein labeled with Cascade Yellow or Pacific Blue was a 5 mm light-emitting diode (LED; LED Supply, catalog No. L3-0-U5TH15-1; Fig. 2 ▸). This LED has a peak wavelength of 400 nm and an emission half-angle of ∼15°. Power to the LED was supplied by a 5 V power supply through a DynaOhm DC resistor module (LED Supply, catalog No. 04006–020) to control the LED current to 20 mA. The LED plugs into a connector and can be easily changed for one with a different wavelength. Alternatively, a 532 nm diode laser was used for excitation of protein crystals labeled with Carboxyrhodamine 6G. The monochromatic laser light was launched into a fiber optic to bring it to the hood, where a cone of light was emitted onto the imaging area. An emission filter suitable for the fluorescent probe to be imaged was inserted between the camera and the clip-on lens.

## 3. Results and discussion   

### 3.1. Magnification lenses   

Several commercially available clip-on macro lenses for smart devices were obtained and evaluated for fluorescent imaging applications. Images with the best overall quality for the cost were obtained using an AUKEY Ora lens with an iPhone 6S, and this lens provided a convenient means of mounting an excitation light source. Modifications to accommodate an excitation source were simple to make. One source of difficulty in using these clip-on lenses was stabilizing them firmly over the center of the camera lens. The more expensive Olloclip Macro Pro Lens for iPhones eliminated the stability difficulties, but introduced new difficulties with the placement of an emission filter.

### 3.2. Fluorescent probes   

**Figure 3:**
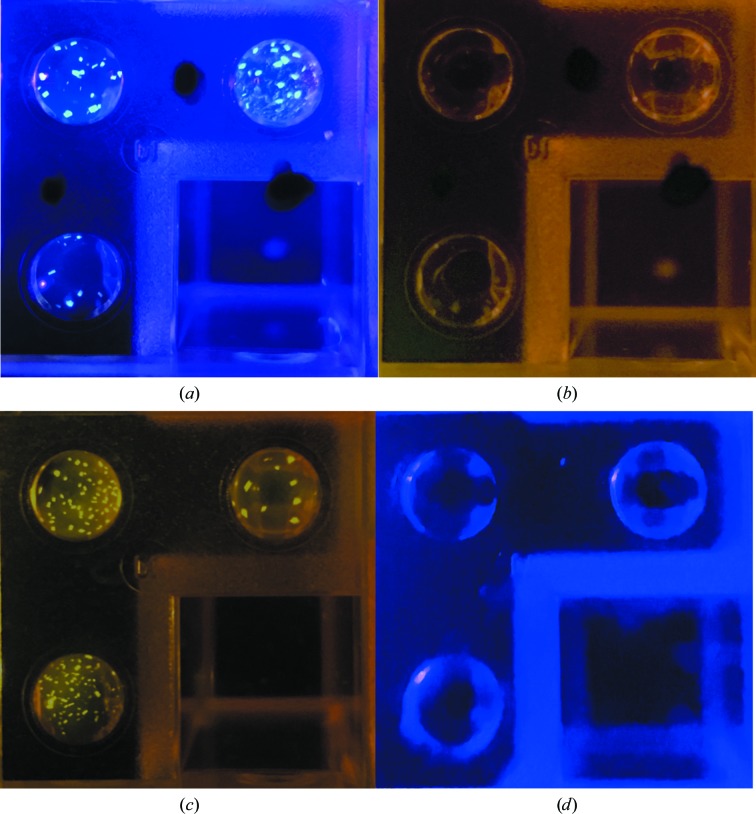
(*a*) Crystals of ConA labeled with the fluorescent probe PB and excited using a 400 nm blue LED without using an emission filter. (*b*) The well shown in (*a*) imaged using a 532 nm green laser diode and a 550 nm high-pass emission filter. (*c*) CR-labeled ConA crystals imaged using a 532 nm green laser diode and a 550 nm high-pass emission filter. (*d*) The well shown in (*c*) imaged under the light from a 400 nm blue LED without an emission filter.

This imaging approach was tested using several fluorescent probes. Crystallization plates containing ConA labeled with PB (excitation and emission at 404 and 455 nm, respectively) were initially imaged through a bandpass filter (Edmund Optics, catalog No. 65-626). Subsequent experiments indicated that the filter was not necessary, resulting in this system having the fewest components and the lowest cost. Protein crystals of ConA TFL with PB were excited with a 400 nm blue LED and imaged without the use of an emission filter (Fig. 3 ▸ *a*). These crystals failed to excite under the 532 nm green diode laser (Fig. 3 ▸ *b*).

The original implementation of this system used CR as the fluorescent probe. The Stokes shift for CR is ∼28 nm (excitation at ∼524 nm, emission at ∼552 nm), the LED peak wavelength was ∼525 nm with a full-width at half-maximum (FWHM) wavelength range of ∼30 nm, and the addition of a 550 nm high-pass emission filter (Edmund Optics, catalog No. 62-977) between the macro lens and the camera lens was required. The wavelength spread of the LED resulted in a significant amount of excitation light still passing through the filter, which obscured the fluorescent signal. However, protein crystals of CR-labeled ConA were detected and imaged using a 550 nm high-pass emission filter and a 532 nm diode laser as an excitation source (Fig. 3 ▸ *c*). The monochromatic laser light was launched into a fiber optic to bring it to the optical port, where a cone of light was emitted onto the imaging area. These crystals failed to fluoresce with the 400 nm blue LED (Fig. 3 ▸ *d*).

Use of this approach is not limited to the fluorescent probes mentioned. LEDs are available with emission peaks sufficiently close to those of most fluorescent probes. Fluorescence visualization is simplified when using probes having a larger Stokes shift, but with some forethought those having a relatively narrow shift can be accommodated, as shown in this work. One important consideration is that fluorescent probes are often very sensitive to their local environment and may undergo absorption and/or emission shifts as a result. However, this may be mitigated by the fact that the probe will be buried in the relatively protected environment of the crystal, potentially precluding effects originating from the bulk solution.

### 3.3. Emission filters   

In the case where the Stokes shift of the fluorescent probe was very narrow, emission filters were needed to prevent reflected excitation light from overwhelming the fluorescent signal. The filters needed to have a very sharp cutoff and a very high blocking optical density at nontransmission wavelengths to minimize reflections obscuring the fluorescence emission. However, the use of a filter more than quadrupled the cost of the system. Two variations of a laser-excitation approach were tried: manually directing a green laser pointer through the translucent hood around the lens and directing the beam into a fiber optic that was passed through a close-fitting aluminium tube through the hood. In both cases the 550 nm high-pass filter was required to block excitation reflections from the camera. While both approaches work, it was felt that switching to a diode laser-excitation source detracted from the goal of an inherently simple low-cost imaging system. However, use of a laser-excitation source does facilitate imaging fluorescence from probes having a very small Stokes shift.

Although some excitation light enters the camera with the filter-free approach used with PB, it is somewhat attenuated by the glass optics of the macro lens and it is readily differentiated from the emission signal. PB has a sufficiently wide Stokes shift that an emission filter was not needed, although one could be used to further enhance the fluorescence over the background. The fluorescent probe CY (excitation at ∼410 nm, emission at ∼560 nm) can also be excited using a 400 nm LED. Owing to the similarities between the CR and CY emission peaks, the 550 nm high-pass filter used with CR was tested with CY (results not shown). As with PB, the use of an emission filter was not necessary for imaging protein crystals labeled with CY. One negative aspect of the use of CY was the fact it is no longer commercially available. The fluorescent probe Pacific Orange (PO; Invitrogen, catalog No. P-30253; excitation at ∼405 nm, emission at ∼551 nm) is commercially available and could be used with the same excitation LED.

### 3.4. Two-color fluorescence   

**Figure 4:**
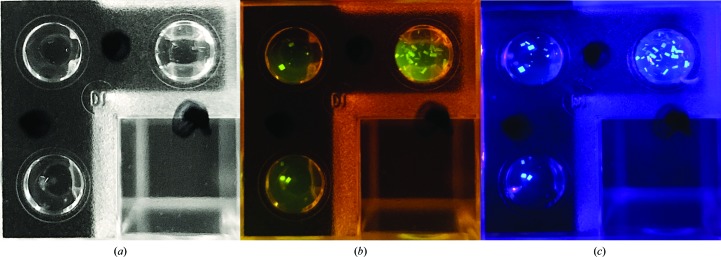
Two-color fluorescence imaging. (*a*) Protein crystals of ConA labeled with the fluorescent probes CR and PB under white light. (*b*) Image of the well shown in (*a*) under the light of a 532 nm green laser diode using a 550 nm high-pass emission filter. (*c*) Image of the well shown in (*a*) illuminated with a 400 nm blue LED with no emission filter.

The method can also be used for two-color fluorescence, which is useful for the visualization and imaging of complexes. This approach required the use of an emission filter. Crystallization screening plates were set up using (i) ConA labeled with PB, (ii) ConA labeled with CR and (iii) a mixture containing 50% PB-labeled ConA and 50% CR-labeled ConA. Screening plates containing CR-labeled ConA were imaged using a 532 nm green diode laser through a fiber optic as the excitation source and a 550 nm emission cutoff filter. Crystallization plates containing PB-labeled ConA were imaged using a 400 nm LED without an emission filter. The screening plate created using a combination of two fluorescent probes was imaged using both sets of optics. A single well containing protein crystals was imaged under white light (Fig. 4 ▸ *a*), the CR optics (Fig. 4 ▸ *b*) and the PB optics (Fig. 4 ▸ *c*).

Other pairs of fluorescent probes may be employed for two-color fluorescence. The ability to distinguish the fluorescence of one probe in the presence of the other is essential. Costs will be higher owing to the required emission filter(s). LEDs typically have a full-width half-maximum wavelength spread of ∼±15°. There is still considerable excitation light relative to the fluorescence signal at the ∼0.1–1% intensity level at the peak fluorescence wavelength for probes with a short Stokes shift. Use of probes with a longer Stokes shift, for example PB and CY, can ameliorate this problem. The other approach, as taken here, is to use a monochromatic light source. While this adds to the complexity, diode lasers can be obtained online at a lower cost than that of emission filters. However, emission filters are still sometimes needed to keep reflected light out of the image.

### 3.5. Proteins   

**Figure 5:**
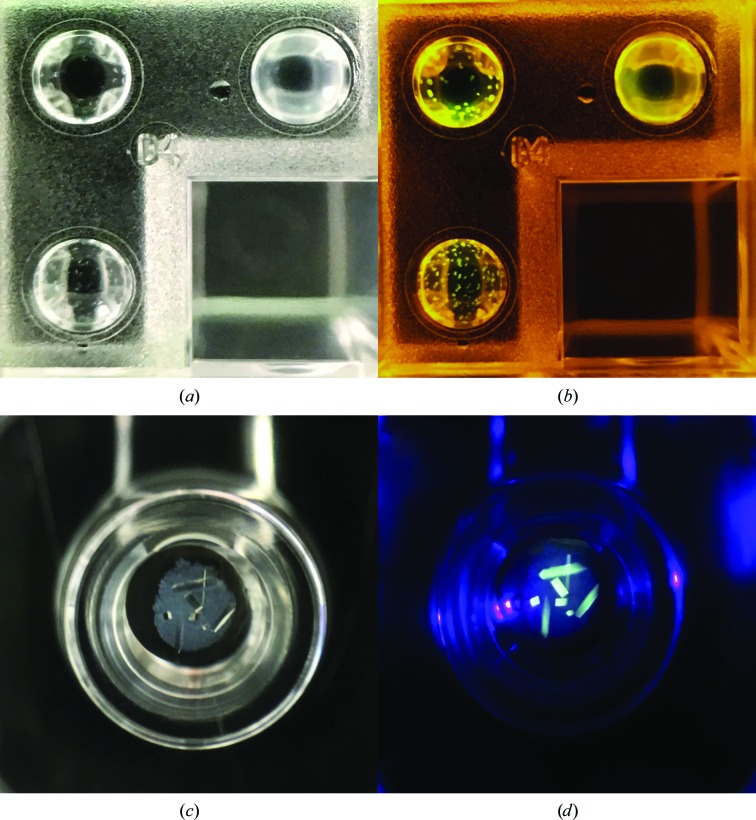
(*a*) Crystals of CR-labeled β-lactoglobulin B under white light. (*b*) The well shown in (*a*) imaged using a 532 nm green laser diode and a 550 nm high-pass emission filter. (*c*) CY-labeled trypsin crystals under white light. (*d*) The well shown in (*c*) imaged under a 400 nm blue LED without an emission filter.

In order to examine this imaging method with additional proteins, crystals of β-lactoglobulin B and trypsin were photographed. β-Lactoglobulin B and trypsin crystals were imaged under white light (Figs. 5 ▸ *a* and 5 ▸ *c*). The same β-lactoglobulin B well was photographed again using the same 532 nm green diode laser, fiber optic and 550 nm emission cutoff filter as used to image CR-labeled ConA crystals (Fig. 5 ▸ *b*). CY-labeled trypsin crystals were imaged using a 400 nm blue LED for excitation without an emission filter (Fig. 5 ▸ *d*).

### 3.6. Imaging   

All fluorescent images were acquired using an iPhone 6S. However, the use of this method is not limited to cell phones. The clip-on lens can be applied to most devices with a camera, but the camera has to be capable of accommodating the macro optics. If image capture is not desired and an emission filter is not needed, then one can bypass the use of a camera. Fluorescing crystals can be observed by manually placing the hood with a mounted LED over a plate of TFL crystals and viewing them under a low-powered microscope (a microscope that is normally used to manually review crystallization plates). Fluorescence from crystals at least as small as 10 µm can be visualized in a dark room and, depending upon the microscopy system, one can zoom in for higher magnification imaging. The room does not have to be totally dark, but it is necessary to remove reflected light from overhead fluorescent lights. In fact, it was discovered that one can directly see CY-labeled crystals without the use of a microscope. However, this is not a practical approach when using probes that require near-UV wavelengths for excitation. If the microscope has a camera port then it can also be used for image capture. Low-cost means of attaching a cell phone to microscopy or other imaging systems are also commercially available.

This method was tested using several imaging devices. The iPhone 6S contained the most versatile imaging system with internal filters, and it provided the best quality images. Several different macro lenses were tested, and the AUKEY Ora lens was found to perform the best at a low cost. The presence of the hood served to reduce background illumination and it was a convenient mounting point for the excitation light. Crystals as small as 10 µm could be imaged, and possibly smaller with the use of higher magnification optics. Other clip-on macro lenses are commercially available and may be more suitable for certain imaging devices.

While not tested, the method of Lukk *et al.* (2016 ▸) may be accommodated by the approach described here. The apparent Stokes shift for excitation at 405 nm is ∼80 nm. The excitation source for their work was a 5 mW laser. If the added cost (and complexity) is acceptable then the LED can be replaced by a 405 nm diode laser, bringing the light into the hood using a fiber optic through the same aluminium tube as used for the LED. However, not all proteins fluoresce. The advantage of the TFL approach over other fluorescence methods is that the fluorescence probe, and thus a signal, will be present. Previous work has clearly shown that the presence of the probe at the target labeling concentration range of 0.1–0.5% will not affect the nucleation rates or quality of the diffraction data obtained (Forsythe *et al.*, 2006 ▸).

### 3.7. Costs   

The cost of a simple system, composed of an imaging lens and LED (Fig. 1 ▸) is less than $50. The cost can be less than $30 depending on the source of the LED, the power supply and how the current-limiting resistor is implemented. The approach taken can potentially be applied to UV fluorescence, but at increased cost driven by the higher price of UV LEDs. Other variations are possible, such as the use of low-cost diode lasers for the excitation source, as demonstrated here for imaging CR-labeled proteins. This approach involves a more complex hardware setup as well as the higher cost of the laser and fiber optic. An emission filter may be required to keep reflected laser light noise out of the signal.

Most automated imaging systems are very expensive. The method of Watts *et al.* (2010 ▸) is closest to our method in cost at less than 1000 Euro. Their approach used the increase in the fluorescence of the dye 1,8-ANS (excitation at ∼360 nm and emission at ∼505 nm) upon diffusing into and binding to the interior of a protein crystal (Groves *et al.*, 2007 ▸). In their implementation, the whole plate was imaged, with each well being excited using a separate LED. The excitation source was directed towards the imaging optics while utilizing both excitation and emission filters. Their design also used crystallization-plate-specific masks to limit the excitation light to only the crystalline-drop positions. Software filtering was used to reduce the background noise. As the method relies upon free probe diffusing into the crystal, it cannot be used for multiple colors. In comparison, the method presented here is a low-cost approach for anyone owning a smart device with a camera. It provides a way to rapidly screen plates containing a wide variety of crystallization solutions and image protein crystals. This approach can also be used for the imaging of complexes, which can save beam time.

Several potential improvements immediately became apparent using this method. When using an autofocusing device, the camera must be securely fixed in position to more easily obtain quality images. A Bluetooth shutter control and tripod were later utilized to address this issue. While these items did stabilize and improve the imaging system, they increased the overall cost. Therefore, all images shown were taken using a handheld iPhone 6S camera while resting the lens hood on the crystallization plate. A second improvement would be an application written for the smart device to adjust the focal point and other exposure parameters, such as filters that may be built into the camera.
